# Longitudinal alterations in brain perfusion and vascular reactivity in the zQ175DN mouse model of Huntington’s disease

**DOI:** 10.1186/s12929-024-01028-3

**Published:** 2024-04-16

**Authors:** Tamara Vasilkovska, Somaie Salajeghe, Verdi Vanreusel, Johan Van Audekerke, Marlies Verschuuren, Lydiane Hirschler, Jan Warnking, Isabel Pintelon, Dorian Pustina, Roger Cachope, Ladislav Mrzljak, Ignacio Muñoz-Sanjuan, Emmanuel L. Barbier, Winnok H. De Vos, Annemie Van der Linden, Marleen Verhoye

**Affiliations:** 1 Bio-Imaging Lab, University of Antwerp, Universiteitsplein 1, 2610 Wilrijk, Antwerp, Belgium; 2 µNEURO Research Centre of Excellence, University of Antwerp, Antwerp, Belgium; 3 Laboratory of Cell Biology and Histology, University of Antwerp, Universiteitsplein 1, 2610 Wilrijk, Antwerp, Belgium; 4 Antwerp Centre for Advanced Microscopy, Universiteitsplein 1, 2610 Wilrijk, Antwerp, Belgium; 5 C.J. Gorter MRI Center, Leiden University Medical Center, Leiden, the Netherlands; 6 Univ. Grenoble Alpes, Inserm, U1216, Grenoble Institut Neurosciences, Grenoble, France; 7 CHDI Management, Inc., the company that manages the scientific activities of CHDI Foundation, Inc, Princeton, NJ USA; 8 Present Address: Takeda Pharmaceuticals, Cambridge, MA USA; 9 Present Address: Cajal Neuroscience Inc, Seattle, WA USA

**Keywords:** Huntington’s disease, Brain perfusion, Cerebral blood flow, Cerebrovascular reactivity, Blood vessels

## Abstract

**Background:**

Huntington’s disease (HD) is marked by a CAG-repeat expansion in the huntingtin gene that causes neuronal dysfunction and loss, affecting mainly the striatum and the cortex. Alterations in the neurovascular coupling system have been shown to lead to dysregulated energy supply to brain regions in several neurological diseases, including HD, which could potentially trigger the process of neurodegeneration. In particular, it has been observed in cross-sectional human HD studies that vascular alterations are associated to impaired cerebral blood flow (CBF). To assess whether whole-brain changes in CBF are present and follow a pattern of progression, we investigated both resting-state brain perfusion and vascular reactivity longitudinally in the zQ175DN mouse model of HD.

**Methods:**

Using pseudo-continuous arterial spin labelling (pCASL) MRI in the zQ175DN model of HD and age-matched wild-type (WT) mice, we assessed whole-brain, resting-state perfusion at 3, 6 and 9 and 13 months of age, and assessed hypercapnia-induced cerebrovascular reactivity (CVR), at 4.5, 6, 9 and 15 months of age.

**Results:**

We found increased perfusion in cortical regions of zQ175DN HET mice at 3 months of age, and a reduction of this anomaly at 6 and 9 months, ages at which behavioural deficits have been reported. On the other hand, under hypercapnia, CBF was reduced in zQ175DN HET mice as compared to the WT: for multiple brain regions at 6 months of age, for only somatosensory and retrosplenial cortices at 9 months of age, and brain-wide by 15 months. CVR impairments in cortical regions, the thalamus and globus pallidus were observed in zQ175DN HET mice at 9 months, with whole brain reactivity diminished at 15 months of age. Interestingly, blood vessel density was increased in the motor cortex at 3 months, while average vessel length was reduced in the lateral portion of the caudate putamen at 6 months of age.

**Conclusion:**

Our findings reveal early cortical resting-state hyperperfusion and impaired CVR at ages that present motor anomalies in this HD model, suggesting that further characterization of brain perfusion alterations in animal models is warranted as a potential therapeutic target in HD.

## Background

Normal brain function requires a continuous nutrient supply to maintain its metabolic and energy demands, for which proper cerebral blood flow (CBF) is crucial [1, 2]. Neurons are in direct communication with components of the neurovascular unit (astrocytes, pericytes, smooth muscle cells) that exert control on the blood vessel tonus [3]. Impairments in these cellular interactions result in an inability to properly coordinate CBF, which then leads to an inefficient distribution of important nutrients to the brain parenchyma, causing neuronal dysfunction [4]. Such cerebrovascular alterations have been implicated as relevant events at different stages in several neurodegenerative disorders, such as Alzheimer’s [5–7] and Huntington’s disease (HD) [8–10].

HD is an autosomal, dominantly inherited neurodegenerative disease with an abnormal expansion of the CAG repeat in the huntingtin gene. This sets in motion a complex cascade of modifications of the mutated huntingtin protein (mHTT) that leads to inevitable neuronal dysfunction and death [11]. Neuronal loss and altered neuronal functioning can yield decreased brain glucose consumption in people with HD (PwHD) [12, 13], which can in turn influence CBF in vulnerable cortical and subcortical brain regions in different stages in HD, before and after clinical motor diagnosis [14–16]. An increasing body of evidence has pointed towards neurovascular uncoupling and regional hypoperfusion as an early and independent event in HD pathology, before brain atrophy occurs [9, 17–19]. A potential underlying cause are changes in cerebrovascular reactivity (CVR), a measure of the capability of the vasculature to autoregulate under subtle physiological stress (e.g., CO_2_), which has been described in two cross-sectional studies in PwHD [10, 20].

Despite the known genetic background, no disease-modifying therapies are available yet, and the identification of validated biomarkers would be very advantageous. Towards this, several animal models that possess different cellular and behavioural HD landmarks have been extensively studied. The heterozygous (HET) form of the zQ175 mice exhibits cellular, behavioural, and cognitive abnormalities, where motor deficits are present by around 6 months onwards, following a temporal progression [21–23].

To better correlate phenotypic alterations with brain perfusion anomalies, we investigated whether alterations in CBF and CVR occur in the zQ175DN HET HD mouse model and, if so, how these alterations change over time, following the phenotypic progression of this model [22, 24].

Brain perfusion can be non-invasively and dynamically (temporal resolution ± 7 s) measured with arterial spin labelling (ASL) MRI [25]. This method uses the endogenous blood-derived water as a diffusible tracer and thereby magnetically tagging these water molecules allows to detect their path through different tissues, by their exchange. Continuous ASL uses a steady-state approach in which, using a long off-resonance RF pulse, the arterial blood is continuously inverted at the level of the carotid arteries. The exchange between the magnetically labelled water in the blood and the brain tissue water leads to a change in the tissue water magnetization. Assuming that water is a freely diffusible tracer, this change of tissue magnetization can directly be correlated with tissue perfusion. A modified version of this method, named pseudo-continuous ASL (pCASL), replaces the long, continuous RF pulse with a continuous train of short RF pulses for labelling that allows for multi-slice acquisition, increased signal-to-noise ratio, and CBF accuracy [26, 27]. A recent cross-sectional study, employing pCASL for the first time in HD, demonstrated decreased CBF in the caudate and putamen in PwHD after clinical motor diagnosis [19].

Using pCASL MRI, we conducted a proof-of-concept study in age-matched zQ175DN HET and wild-type (WT) mice, assessing resting-state CBF at an advanced age of 13 months and CVR using a hypercapnic challenge at the age of 15 months. This was followed by two longitudinal studies at different ages: 3, 6 and 9 months for the resting-state CBF and 4.5, 6 and 9 months for the CVR assessment. Moreover, to better understand the underlying vascular changes that could drive the perfusion alterations, we used immunofluorescence to investigate age-dependent changes in vessel density and length at 3, 6, 8, 12 and 14 months of age in 8 brain regions shown to be affected in PwHD. We hypothesized that age-dependent, cortical, and striatal perfusion and reactivity alterations would be present in this HD mouse model.

## Materials & methods

### In-vivo pCASL study

#### Animals

**Fig. 1:**
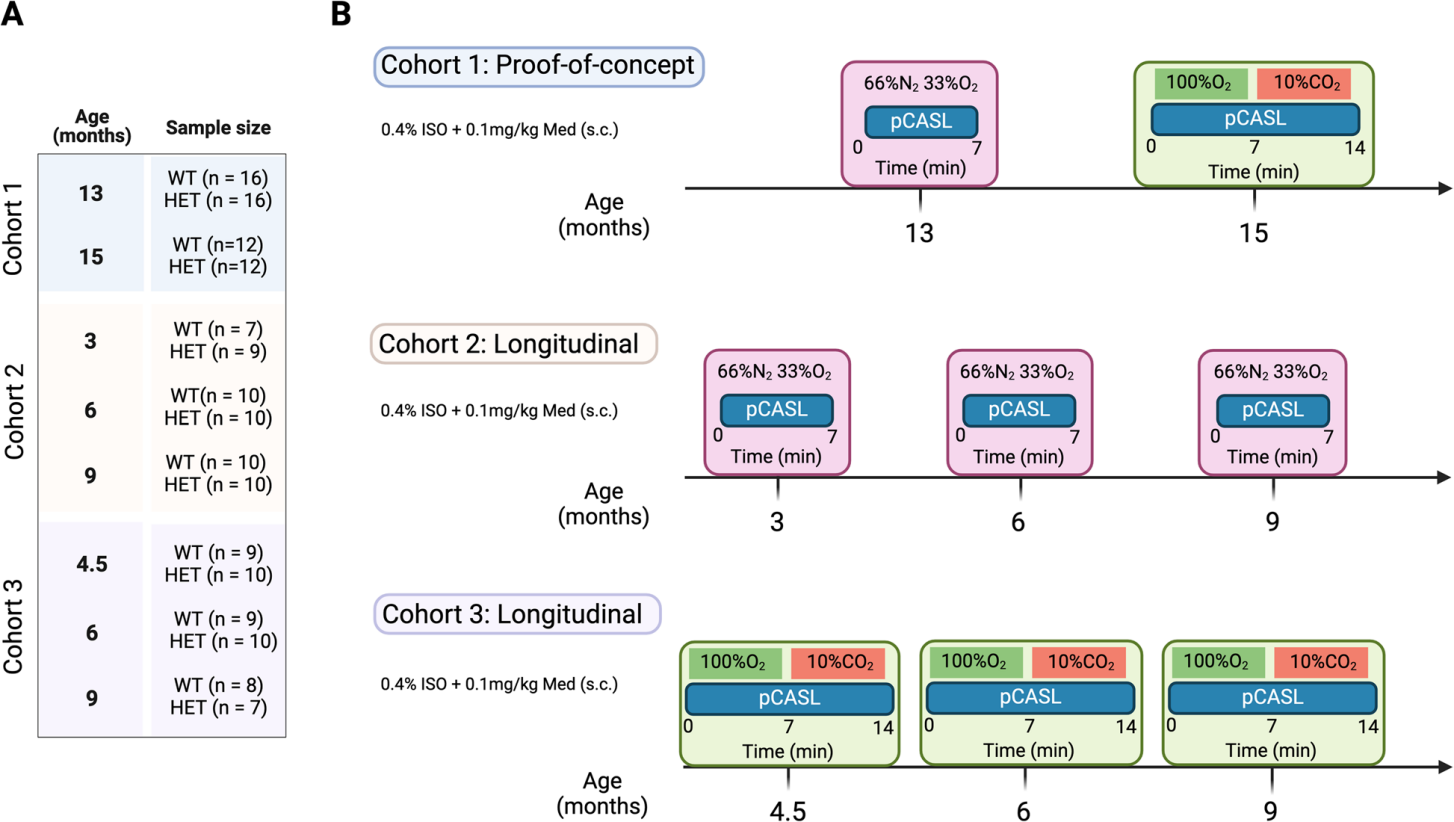
Experimental design. (**A**) Sample sizes of WT and zQ175DN HET groups for each age and cohort (**B**) Experimental design for each cohort, for the proof-of-concept cohort 1, and the longitudinal studies: cohort 2 – resting-state CBF and cohort 3—CVR

In this study, HET zQ175DN KI and WT [28] age-matched littermates (C57BL/6 J background, CHDI-81003019, JAX stock #029928), were used. The zQ175DN KI (without a floxed neomycin cassette) mouse model has the human HTT exon 1 substitute for the mouse *Htt* exon 1 with ~ 180–220 CAG repeats long tract. This model is a modified version of the zQ175 KI [23] where the congenic C57BL6J is used as the strain background [29]. The first motor deficits are observed at 6 months, marked as an onset of phenoconversion [22]. The HET form of zQ175DN has a slow progression reflected in the increase of mHTT aggregation from 3 until 12 months, initially appearing in the striatum at 3 and later in the cortex at 8 months of age [24]. A total of 36 HET zQ175DN KI and 35 WT age-matched littermates were used, distributed over 3 cohorts (Fig. 1A). We performed a proof-of-concept (PoC) study in cohort 1 for resting-state perfusion (13 months of age) and CVR (15 months of age), followed by two longitudinal pCASL studies where we assessed either resting-state perfusion or CVR: cohort 2 for resting-state perfusion (ages 3, 6 and 9 months of age) and cohort 3 for CVR (ages 4.5, 6 and 9 months of age (Fig. 1B). In all experiments, mice were initially anaesthetized with 2% isoflurane (Isoflo®, Abbot Laboratories Ltd., USA) in oxygen-enriched (66% N_2_ and 33% O_2_) for the plain perfusion studies [30], and for the CVR studies, 100% O_2_ during the baseline condition and 90% O_2_ + 10% CO_2_ [31] during the hypercapnic condition. The gas combinations were kept the same throughout the whole experiment. After the animal was positioned in the scanner, a subcutaneous bolus injection of medetomidine (0.05 mg/kg; Domitor, Pfizer, Karlsruhe, Germany) was applied followed by a gradual decrease of isoflurane to 0.4% over the course of 30 min which was kept at this level for the duration of the experiment. Meanwhile, a continuous subcutaneous infusion of medetomidine (0.1 mg/kg/h), starting 10 min post bolus medetomidine injection, was applied in combination with the isoflurane. The anaesthesia protocol used in this study is an established combination used for rodent resting-state fMRI [32, 33]. Throughout the experiment, all physiological parameters (breathing rate, heart rate, O_2_ saturation, and body temperature) were continuously monitored to ensure stable conditions. Animals were single-housed in individually ventilated cages with food and water ad libitum and continuous monitoring for temperature and humidity under a 12 h light/dark cycle. The animals were kept in the animal facility for at least a week to acclimatize to the current conditions before the experimental procedures.

#### Image acquisition

MRI scans were acquired on a 7 T Pharmascan MR scanner with a 16 cm diameter horizontal bore, a quadrature 70 mm whole body resonator as a transmit coil and a 4 channel receive-only surface coil (Bruker, Germany). After positioning, three orthogonal T2-weighted Rapid Acquisition with Refocused Echoes (RARE) anatomical reference images (TR = 2000 ms, TE = 33 ms, matrix dimensions (MD) = (256 × 256), field of view (FOV) = (20 × 20) mm^2^, 12 slices) were acquired to enable a consistent slice package position for the pCASL scans for all subjects/time points. A time-of-flight angiogram was acquired to define the labelling position. Preceding the pCASL acquisition, two pre-scans were performed to optimize the phase of the pCASL label and control pulses [34]. The optimal label and control phases were subsequenlty used in all pCASL scans (i.e. in the perfusion scans of resting state and hypercapnic condition,, as well as in the labelling efficiency scan). Control and label images were acquired using the pCASL sequence [34] during which labelling pulses were applied in the neck (~ 8–11 mm caudal to the central imaging plane) with a duration of ($\uptau )$ 3000 ms followed by a 200 ms post-labelling delay (PLD) and a single-shot spin echo planar imaging (SE-EPI) acquisition (TR = 3450 ms, TE = 19.5 ms, MD (96 × 64), FOV (25 × 25) mm^2^, spatial resolution (260 × 400) µm^2^, 5 slices of 0.8 mm slice thickness. To measure resting-state perfusion, 120 (60/60 control/label) images were acquired for 7 min. For the CVR, a baseline condition of 120 (60/60 control/label) images were acquired with a duration of 7 min, and from 7 until 14 min a CO_2_ challenge was administered and additional 120 images were acquired in that hypercapnic period (Fig. 1B). To calculate the labelling efficiency, α, a single (4 averages) label/control pCASL-scan with a flow compensated FLASH readout (labelling time of 200 ms without a PLD) is acquired, for one imaging slice located downstream the labelling plane, 4 mm caudal from the imaging volume. As the quantification of absolute CBF requires regional T1-values, additional non-selective inversion recovery (IR) scans were acquired with 23 inversion times (TI) ranging from 15 to 5000 ms and TR = 10 s, with identical SE-EPI parameters as the pCASL scan (total scan time 4 min 30 s). The total scan time for each experiment was approximately 1 h and 30 min.

#### Image processing

T1 maps are calculated by the non-linear fitting of the T1-relaxation equation for inversion recovery: S(TI) = A + B exp(-TI/T1) to the signal intensities for the different TI, S(TI). Control and label images were realigned to the first scan, using a 6-parameter rigid-body spatial transformation estimated with the least-squares approach. Next, the individual IR image with TI = 5 s was co-registered to the mean image of the pCASL-scan, and the estimated transformation was applied to the T1 map. The absolute CBF-maps were calculated voxel-wise for each repetition (control/label pair of images) and slice, using the one-compartment model of Buxton [35] as shown in the following equation:

$$CBF=\frac{\lambda \cdot\Delta S\cdot exp\left(\frac{PLD}{{T}_{{1}_{blood}}}\right)}{2\cdot \alpha \cdot {T}_{1}^{\mathrm{^{\prime}}}\cdot {S}_{control}\cdot \left(1-{\text{exp}}\left(\frac{-\tau }{{T}_{1}^{\mathrm{^{\prime}}}}\right)\right)}$$

Here, $\lambda$ is the blood–brain partition coefficient (0.89 ml/g), $\Delta S$ is the magnetization difference (signal intensity) between control and label images using the surround subtraction method, ${T}_{{1}_{blood}}$ is the T1 relaxation time constant of the arterial blood (1700 ms at 7 T), with α as the calculated labelling efficiency and T_1_’ the estimated T1 relaxation time constant. To account for variations in arterial transit time across multiple slices, effective PLD (PLD_eff_) was calculated, using an oscilloscope, given by the following equation:

$$PL{D}_{eff}\left(slic{e}_{number}\right)=PLD+7ms+\left(slic{e}_{number}-1\right) x \frac{TR- \tau -PLD }{\# slices}$$

For the first slice, 7 ms needs to be added to the PLD because of a fat suppression gradient applied just before the image acquisition of this slice. The time remaining is then equally divided over the number of slices. To spatially normalize the CBF maps, we created a study-based EPI template from all subjects’ averaged EPI images for each time point in the PoC study and a 1^st^-time point-based template for each longitudinal study. Based on these estimated transformation parameters, all CBF repetitions per subject were normalized to the study-specific template. In-plane smoothing was applied to these normalized CBF maps using a Gaussian kernel with full width at half maximum of twice the voxel size. CBF maps were further averaged over repetitions per condition (baseline/CO_2_ challenge) to calculate the individual CVR, where % ΔCBF was obtained by using the following formula: ((CBF_Challenge_ – CBF_Baseline_)/CBF_Baseline_) *100. Mice with bad labelling efficiency or T1 map were excluded from further analysis and the final sample sizes used are shown in Fig. 1A.

The above steps were performed using Statistical Parametric Mapping (SPM) using SPM12 (Wellcome Centre for Human Neuroimaging, London, UK). Template creation was done using Advanced Normalization Tools (ANTs).

#### Analysis

Two types of analysis were performed to assess differences between genotypes in perfusion and CVR. First, an exploratory voxel-based (VBA) and second, a region-based analysis (RBA). For the RBA, parcels that encompass the region of interest (ROI) bilaterally, were manually delineated for each slice on the corresponding study-based template. The regions were based on previous studies in this model that have shown functional alterations [36, 37]. These included: retrosplenial cortex (RspCtx), cingulate cortex (CgCtx), somatosensory cortex 1/2 (S1/2Ctx), motor cortex 1/2 (M1/2Ctx), caudate putamen (CPu), piriform cortex (PiriCtx), insular cortex (InsCtx), thalamus (Thal), globus pallidus (GP), Visual cortex 1/2 (V1/2Ctx), auditory cortex (AuCtx) and claustrum (Cl), defined based on the Paxinos and Franklin’s mouse brain atlas, corresponding to 5 coronal Bregma levels: + 1.34 mm, + 0.26 mm, -0.46 mm, -1.82 mm and -2.30 mm. For each subject, at each age and condition, the mean CBF value for every region at different Bregma levels was extracted from the respective mean CBF map. Mean regional CVR values are extracted from the CVR map.

#### Ex-vivo immunofluorescence (IF) study

To assess blood vessel alterations, an IF study was performed on 60 age-matched, male WT (*n* = 6) and zQ175DN HET (*n* = 6) mice at 3, 6, 8,12 and 14 months of age for 8 brain regions: the Caudate putamen, medial (mCPu) and lateral portion (lCPu), CgCtx, InsCtx, PiriCtx, S1Ctx, M1Ctx and M2Ctx. Blood vessels were visualized with Lectin-Dylight 488 (1:25, Vector Laboratories, Cat. # DL-1174) and sections were imaged with a Perkin Elmer Ultraview Vox dual spinning disk confocal microscope, mounted on a Nikon Ti body using a 40 × dry objective (numerical aperture 0.95). Details regarding the brain preparation, IF protocol and imaging can be found in Vasilkovska et al [38]*.*

#### Image analysis

A macro script was written for FIJI image analysis [39] and is available on github (https://github.com/DeVosLab/Huntington). Blood vessels were detected in maximum projections of 30 µm z-stacks. After background subtraction, a multi-scale tubeness filter [40] was applied to enhance both small and large vessel structures in the image. A user-defined threshold was used to segment the blood vessels in this enhanced image. The total area of these regions was quantified and reported as the projected blood vessel area. Skeletonization was performed on the obtained vessel masks after which the length of the main axis (defined as the longest path along the vessel) in the FOV was quantified. Data analysis was performed in R [41].

### Statistical analysis

In the pCASL study, for the VBA, two-sample T-tests were performed to assess genotypic differences for each condition and each age (FDR corrected, *p* < 0.05, cluster size (k) ≥ 10). In the RBA, genotype differences were assessed for all ROIs per slice (multiple two-sample t-tests, FDR corrected, *p* < 0.05). In the longitudinal studies, to assess the temporal evolution of CBF and CVR changes for specific ROIs, a mixed effects model for repeated measures was applied with main factors of genotype and age and genotype*age interaction. In the case of interaction, post hoc comparisons (FDR corrected, *p* < 0.05) were performed for values within each genotype across ages compared to a control age of 3 months and between genotypes per age. When no interaction was present, the model was recalculated only for the main effects and a post hoc comparison (FDR, *p* < 0.05) was performed for each effect separately.

In the IF study, image-based outlier detection was applied using the median ± 3 × median absolute deviation as outer limits within each brain region and animal ID. If fewer than 3 images were retained, the animal ID was discarded from further analysis for the specific region. In the next step, all variables were averaged for each animal ID and region after which a similar, ID-based outlier detection was applied. For longitudinal assessment of all markers, two-way ANOVA was used with main factors of genotype and age and genotype*age interaction. In the case of an interaction, the same post hoc comparisons were applied as for the mixed effects model in the RBA analysis of the pCASL study. When no interaction was present, the model was recalculated only for the main effects and a post hoc comparison (FDR, *p* < 0.05) was performed for each effect separately (for age, comparisons are made to 3 months as a control). Outlier detection was performed in R. All statistical analyses and graph visualizations were performed using GraphPad Prism (version 9.4.1 for Windows, GraphPad Software, San Diego, California USA, www.graphpad.com).

## Results

### Alterations in brain perfusion in zQ175DN mice

**Fig. 2:**
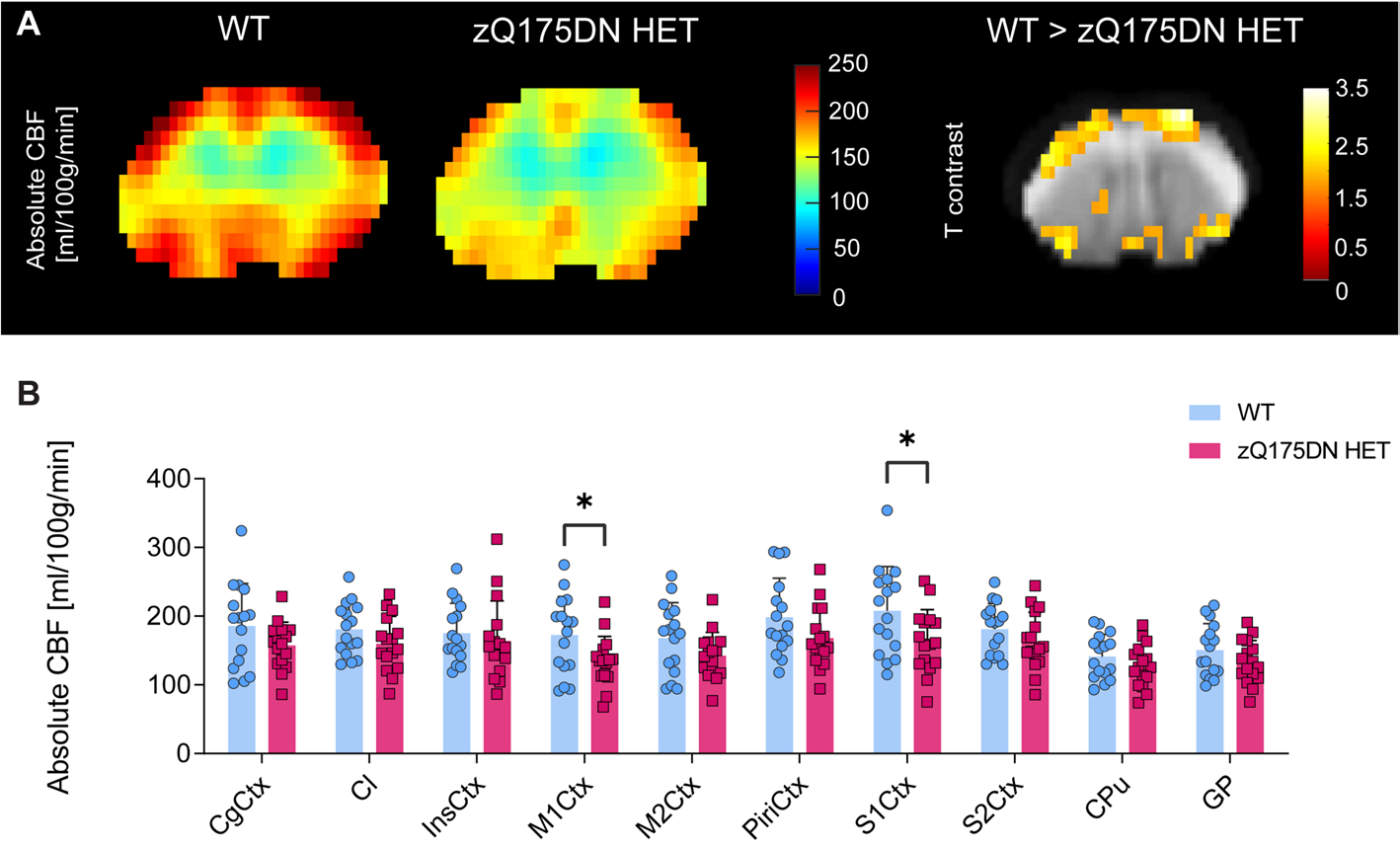
Brain perfusion differences in the zQ175DN HET mice at 13 months of age. (**A**) Mean midbrain absolute CBF maps in both WT and zQ175DN HET group and a statistical T-map from VBA, overlaid on the study-specific template, showing local differences between genotypes (Two-sample T-test, *p* < 0.05, uncorrected, k ≥ 10); (**B**) RBA in predefined cortical and subcortical regions, represented with subject values of absolute CBF and a group mean ± SD (multiple two-sample T-test, *p* < 0.05, uncorrected); CgCtx—Cingulate Cortex, Cl – Claustrum, InsCtx – Insular Cortex, M1/2Ctx – Motor Cortex 1/2, S1/2Ctx – Somatosensory Cortex 1/2, CPu – Caudate Putamen, GP – Globus Pallidus

To assess whether CBF changes exist in this model, we performed both VBA and RBA in the zQ175DN HET and WT mice at 13 months of age. VBA analysis revealed decreased absolute CBF in several cortical regions, such as the M1Ctx, M2Ctx and the S1Ctx, in the zQ175DN HET group as compared to WT (*p* < 0.05, uncorrected; Fig. 2A). In addition, RBA confirmed these results, where we found decreased CBF in the same cortical regions (*p* < 0.05, uncorrected; Fig. 2B).

**Fig. 3:**
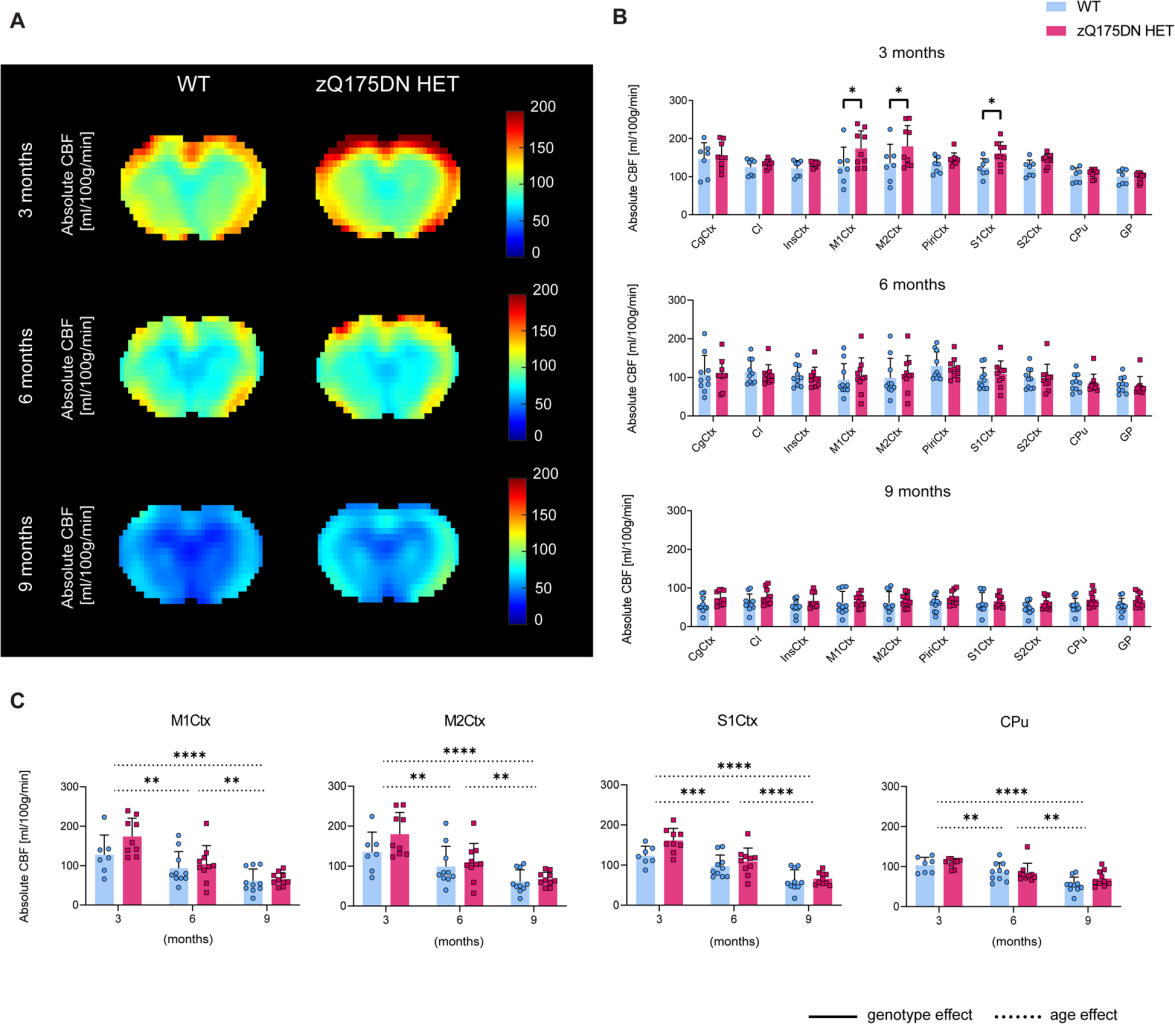
Early and transient perfusion alterations in the zQ175DN HET mice. (**A**) Mean midbrain absolute CBF maps in both WT and zQ175DN HET group at 3, 6 and 9 months of age; (**B**) RBA in predefined cortical and subcortical regions at 3, 6 and 9 months, represented with subject values of absolute CBF and a group mean ± SD (multiple two-sample T-test, *p* < 0.05, FDR corrected); (**C**) Longitudinal assessment of absolute CBF differences between genotypes in four regions (mixed effect model, *p* < 0.05); Significant difference after FDR correction * *p* ≤ 0.05, ** *p* ≤ 0.01, *** *p* ≤ 0.001, **** *p* ≤ 0.0001; CgCtx—Cingulate Cortex, Cl – Claustrum, InsCtx – Insular Cortex, M1/2Ctx – Motor Cortex 1/2, S1/2Ctx – Somatosensory Cortex 1/2, CPu – Caudate Putamen, GP – Globus Pallidus

The differences observed at 13 months, motivated the investigation of changes in CBF at earlier time points in the zQ175DN HET model. The exploratory VBA, which was performed to obtain a global overview of the absolute CBF (Fig. 3A), revealed no significant genotypic differences at any age (*p* < 0.05, FDR corrected). However, the RBA showed, already at 3 months, significantly increased CBF in the zQ175DN HET group in M1Ctx, M2Ctx and S1Ctx as compared to WT (*p* < 0.05, FDR corrected; Fig. 3B). No significant genotypic differences were present in any region at 6 and 9 months of age. In addition, we investigated the longitudinal changes for the three cortical regions that showed alterations at 3 months together with the CPu, a key affected region in HD. In all regions no significant interaction but a significant age effect was found, demonstrating a gradual decrease of absolute CBF with age in both groups (Fig. 3C).

### CVR impairments in zQ175DN mice

**Fig. 4:**
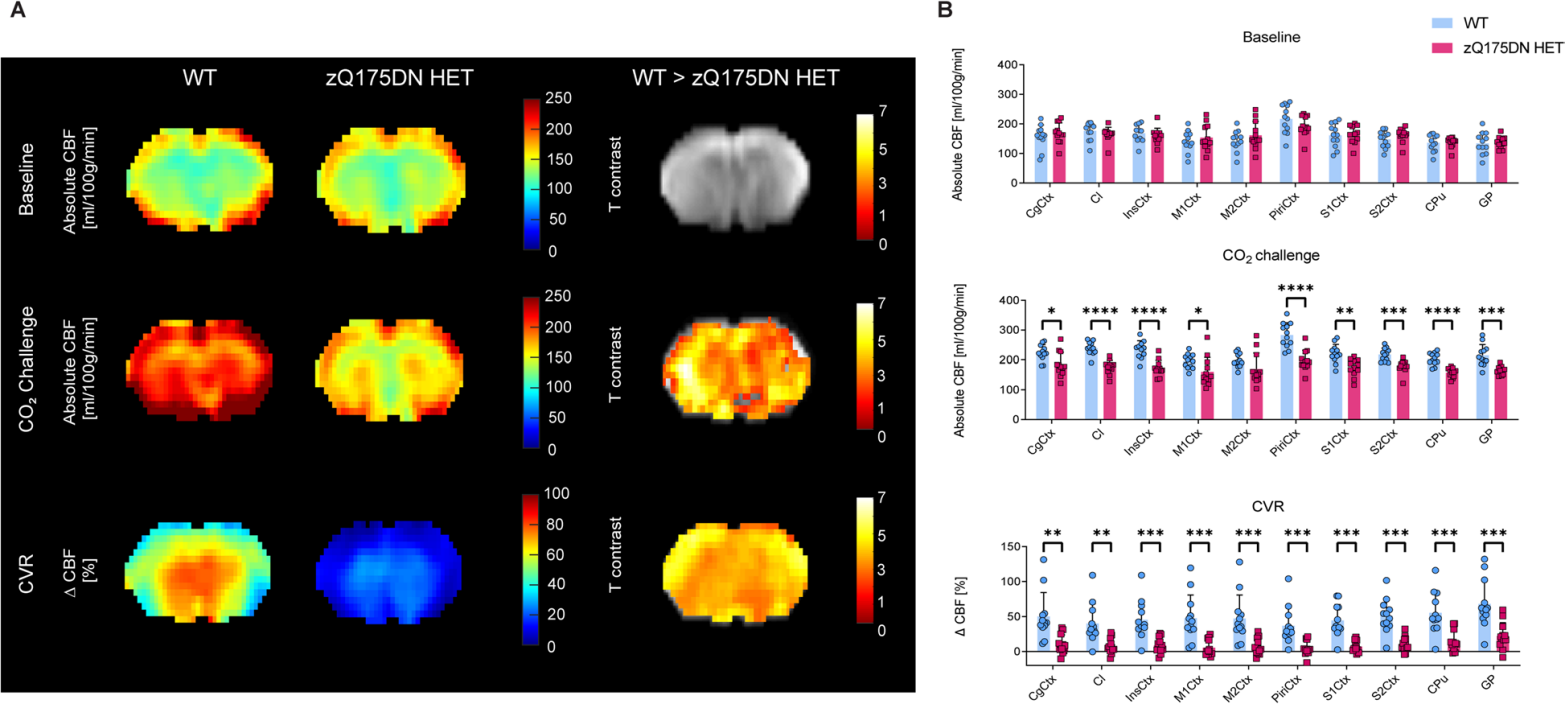
Altered hypercapnic CBF and CVR in zQ175DN HET mice at 15 months of age. (**A**) Mean midbrain absolute CBF maps under baseline, under CO_2_ Challenge and CVR in both WT and zQ175DN HET group and a statistical T-map from VBA for each condition, overlaid on the study-specific template, showing local genotypic differences (Two-sample T-test, *p* < 0.05, FDR corrected, k ≥ 10); (**B**) RBA in predefined cortical and subcortical regions under baseline, CO_2_ Challenge and CVR, represented with subject values of absolute CBF and a group mean ± SD (multiple two-sample T-test, *p* < 0.05, FDR corrected); Significant difference after FDR correction * *p* ≤ 0.05, ** *p* ≤ 0.01, *** *p* ≤ 0.001, **** *p* ≤ 0.0001; CgCtx—Cingulate Cortex, Cl – Claustrum, InsCtx – Insular Cortex, M1/2Ctx – Motor Cortex 1/2, S1/2Ctx – Somatosensory Cortex 1/2, CPu – Caudate Putamen, GP – Globus Pallidus

To examine whether vascular reactivity – induced CBF alterations are present, we performed a hypercapnia PoC study at 15 months of age in the zQ175DN HET mice. VBA analysis revealed no differences between genotypes under baseline conditions, but a significant globally lowered CBF under CO_2_ challenge and a global reduction of CVR in the zQ175DN HET group as compared to WT (*p* < 0.05, FDR corrected; Fig. 4A). The RBA confirmed these genotypic differences, where a significant reduction in CBF under challenge and ∆CBF were present in all ROIs except for M2Ctx (*p* < 0.05, FDR corrected; Fig. 4B).

**Fig. 5:**
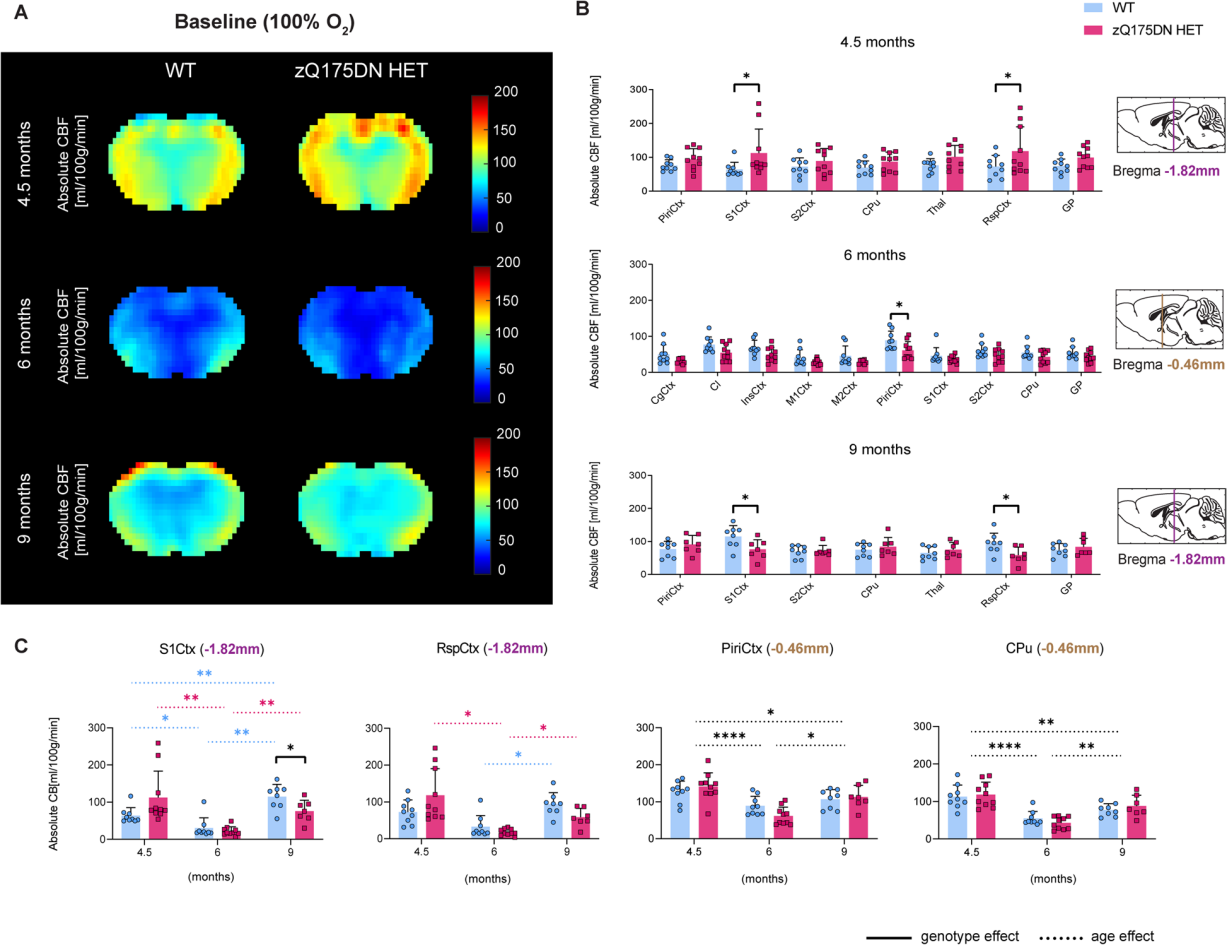
CBF alterations under baseline (100% O_2_) conditions in the zQ175DN HET mice. (**A**) Mean midbrain absolute CBF maps in both WT and zQ175DN HET group at 4.5, 6 and 9 months of age; (**B**) RBA in predefined cortical and subcortical regions at 4.5, 6 and 9 months at different Bregma levels, represented with subject values of absolute CBF and a group mean ± SD (multiple two-sample T-test, *p* < 0.05, FDR corrected); (**C**) Longitudinal assessment of absolute CBF genotypic changes in four regions (mixed effect model, *p* < 0.05); Dashed colored lines represent the overall age effect within WT (blue) and zQ175DN HET (red). Significant difference after FDR correction * *p* ≤ 0.05, ** *p* ≤ 0.01, *** *p* ≤ 0.001, **** *p* ≤ 0.0001; CgCtx—Cingulate Cortex, RspCtx – Retrosplenial Cortex, Cl – Claustrum, InsCtx – Insular Cortex, Piri – Piriform Cortex, M1/2Ctx – Motor Cortex 1/2, S1/2Ctx – Somatosensory Cortex 1/2, CPu – Caudate Putamen, GP – Globus Pallidus, Thal—Thalamus

As CVR alterations were a prominent change at a late age, we aimed to investigate when the starting point of these impairments is, and thus we assessed reactivity changes in earlier ages (4.5, 6 and 9 months of age) in the zQ175DN HET and WT mice. The VBA revealed no significant differences between genotypes under any of the conditions (baseline, CO_2_ challenge, CVR) at any age (*p* < 0.05, FDR corrected). RBA revealed heterogeneous CBF differences, which were Bregma level dependent. At 4.5 months of age, at Bregma -1.82 mm, there was a significantly increased CBF in S1Ctx and RspCtx in the zQ175DN HET group, while the same regions showed a significant decrease in perfusion at 9 months of age (*p* < 0.05, FDR corrected; Fig. 5B). At 6 months, a CBF reduction was only present in the PiriCtx, at Bregma -0.46 mm. Longitudinal assessment of these regions revealed a significant interaction between age and genotype in both the S1Ctx (*p* = 0.006) and RspCtx (*p* = 0.012). In the S1Ctx, the difference between genotypes was present only at 9 months with decreased CBF in the zQ175DN HET (Fig. 5C). Within each genotype, there was a significant decrease between 4.5 and 6 and an increase between 6 and 9 months of age, however, a significant CBF increase from 4.5 to 9 months was only present in the WT group (Fig. 5C). Post-hoc comparisons of the RspCtx showed within each genotype, age-dependent changes where both groups showed a significant increase in CBF from 6 to 9 months of age, while only the zQ175DN HET exhibited a significant decrease of CBF from 4.5 to 6 months. The PiriCtx and the CPu showed only a main age effect, with the same age-dependent trend in both groups, as observed in the other regions (Fig. 5C).

**Fig. 6:**
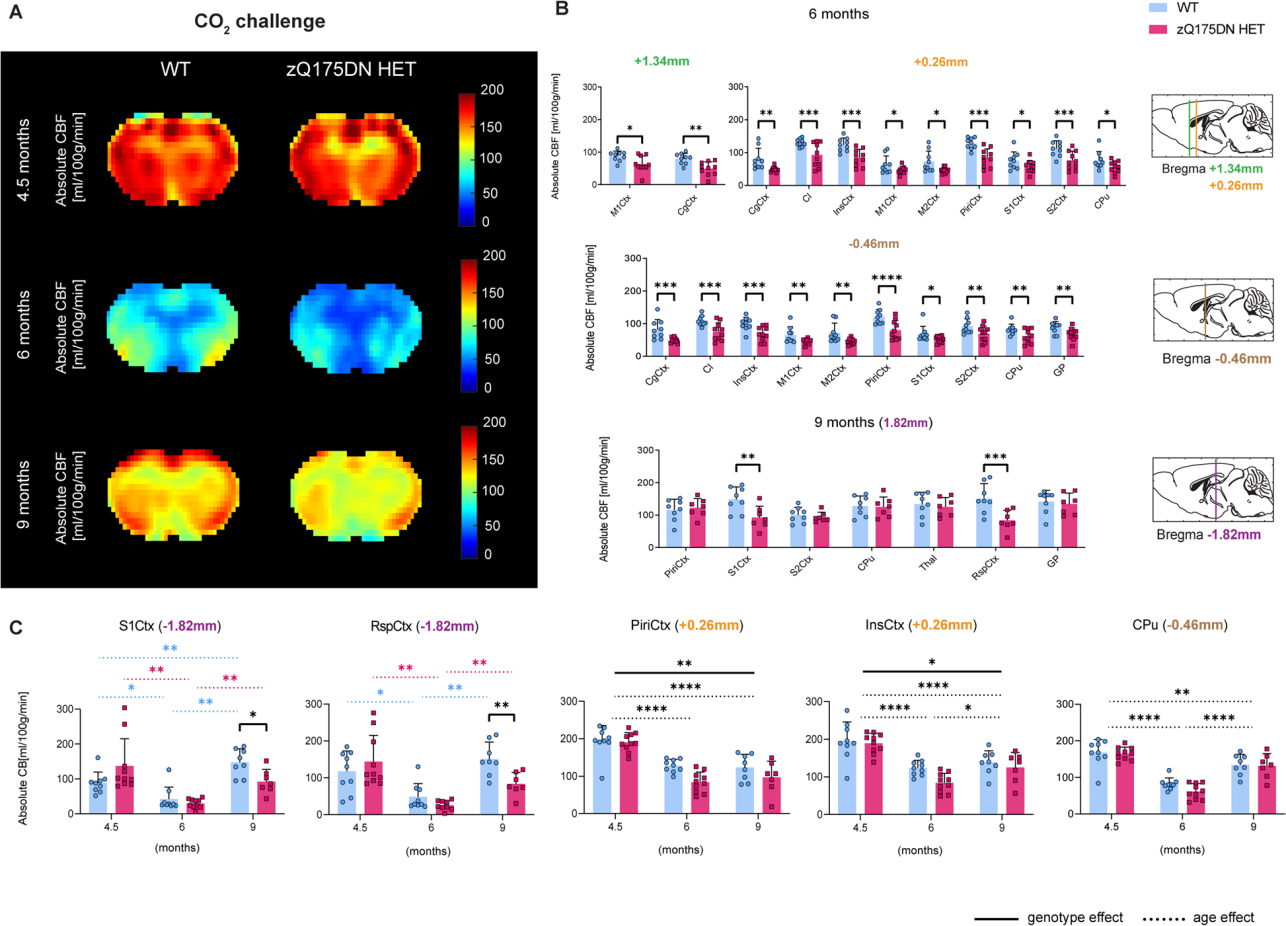
CBF alterations under hypercapnia (10% CO_2_ challenge) in the zQ175DN HET mice. (**A**) Mean midbrain absolute CBF maps in both WT and zQ175DN HET group at 4.5, 6 and 9 months of age; (**B**) RBA in predefined cortical and subcortical regions at 6 and 9 months at different Bregma levels, represented with subject values of absolute CBF and a group mean ± SD (multiple two-sample T-test, *p* < 0.05, FDR corrected); (**C**) Longitudinal assessment of absolute CBF genotypic changes in five regions (mixed effect model, *p* < 0.05); Dashed colored lines represent the overall age effect within WT (blue) and zQ175DN HET (red). Significant difference after FDR correction * *p* ≤ 0.05, ** *p* ≤ 0.01, *** *p* ≤ 0.001, **** *p* ≤ 0.0001; CgCtx—Cingulate Cortex, RspCtx – Retrosplenial Cortex, Cl – Claustrum, InsCtx – Insular Cortex, Piri – Piriform Cortex, M1/2Ctx – Motor Cortex 1/2, S1/2Ctx – Somatosensory Cortex 1/2, CPu – Caudate Putamen, GP – Globus Pallidus, Thal—Thalamus

Under hypercapnia (10% CO_2_ challenge), an overall CBF increase occurs, as observed from the average mean CBF group maps (Fig. 6A). The RBA revealed changes at 6 months, where an overall CBF reduction in multiple regions across different Bregma levels was present in the zQ175DN HET group (*p* < 0.05, FDR corrected; Fig. 6B). At 9 months of age, only significantly reduced CBF was present in the S1Ctx and RspCtx at Bregma -1.82 mm. Longitudinal changes revealed an age and genotype interaction in S1Ctx (*p* = 0.0049) and RspCtx (*p* = 0.0203), with a difference between genotypes present only at 9 months of age, while age-dependent changes within each group followed the same trend as observed under baseline conditions (Fig. 6C). The PiriCtx and InsCtx showed no interaction but a significant age and genotype effect, with an overall decreased absolute CBF in the zQ175DN HET group compared to WT. The CPu had only a significant age effect with the same trend of a significant decrease from 4.5 to 6 and 9 months and an increase from 6 to 9 months of age.

**Fig. 7:**
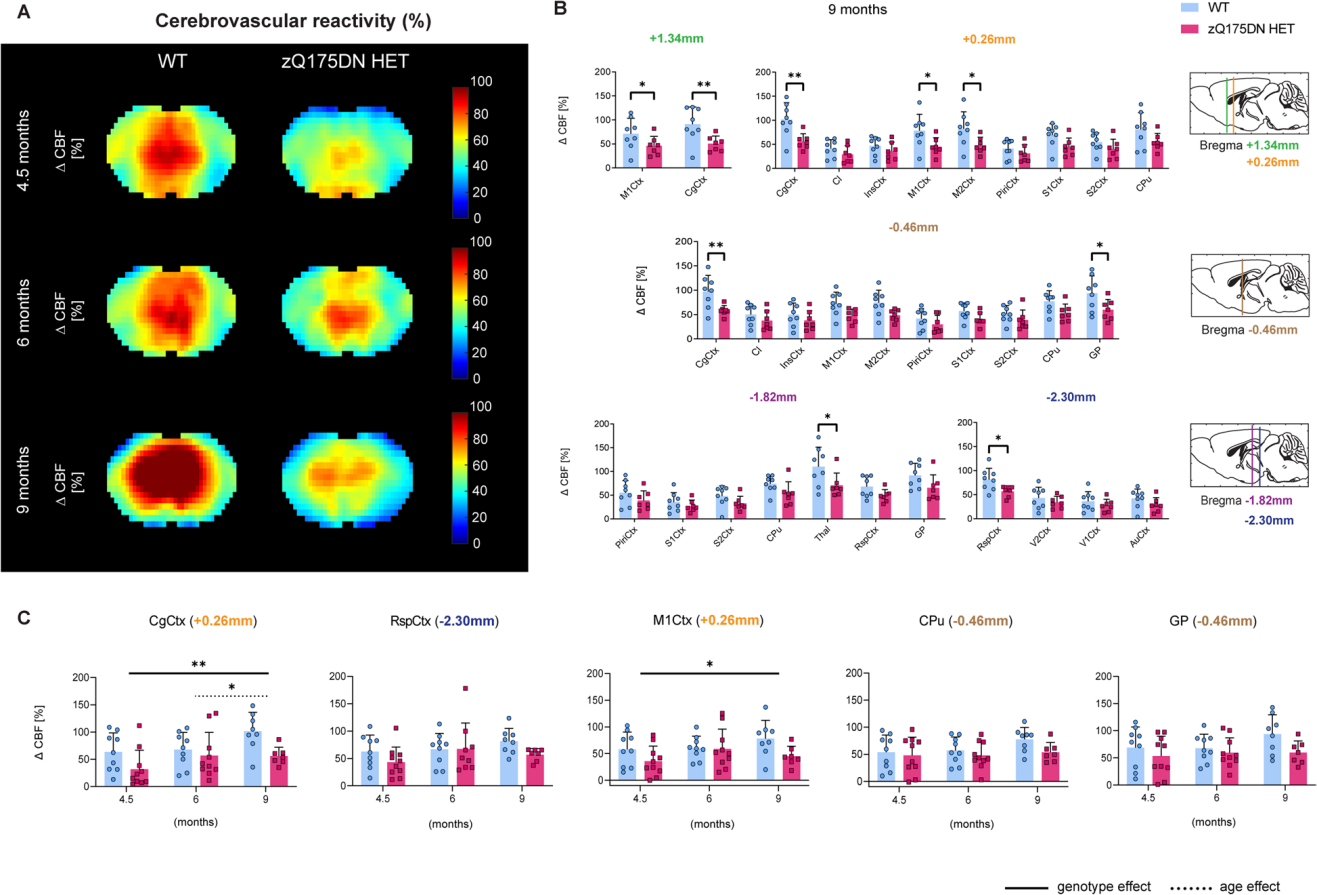
CVR impairments in the zQ175DN HET mice. (**A**) Mean midbrain ∆CBF (%) maps in both WT and zQ175DN HET groups at 4.5, 6 and 9 months of age; (**B**) RBA in predefined cortical and subcortical regions at 9 months at different Bregma levels, represented with subject values of ∆CBF (%) and a group mean ± SD (multiple two-sample T-test, *p* < 0.05, FDR corrected); (**C**) Longitudinal assessment of ∆CBF (%) genotypic changes in five regions (mixed effect model, *p* < 0.05); Significant difference after FDR correction * *p* ≤ 0.05, ** *p* ≤ 0.01, *** *p* ≤ 0.001, **** *p* ≤ 0.0001; CgCtx—Cingulate Cortex, RspCtx – Retrosplenial Cortex, Cl – Claustrum, InsCtx – Insular Cortex, Piri – Piriform Cortex, M1/2Ctx – Motor Cortex 1/2, S1/2Ctx – Somatosensory Cortex 1/2, CPu – Caudate Putamen, GP – Globus Pallidus, Thal—Thalamus

Finally, we assessed whether impairments in CVR are present at these ages (Fig. 7A). RBA revealed that CVR is significantly reduced only at 9 months of age and in several regions across different Bregma levels amongst which are the M1Ctx, CgCtx, M2Ctx, GP, Thal and RspCtx (*p* < 0.05, FDR corrected; Fig. 7B). No genotype and age interaction were found in the longitudinal assessment of any of the ROIs, however, a genotype effect with an overall decreased CVR in the zQ175DN HET group was present in the CgCtx and M1Ctx (Fig. 7C).

### Blood vessel changes in the zQ175DN HET mice

**Fig. 8:**
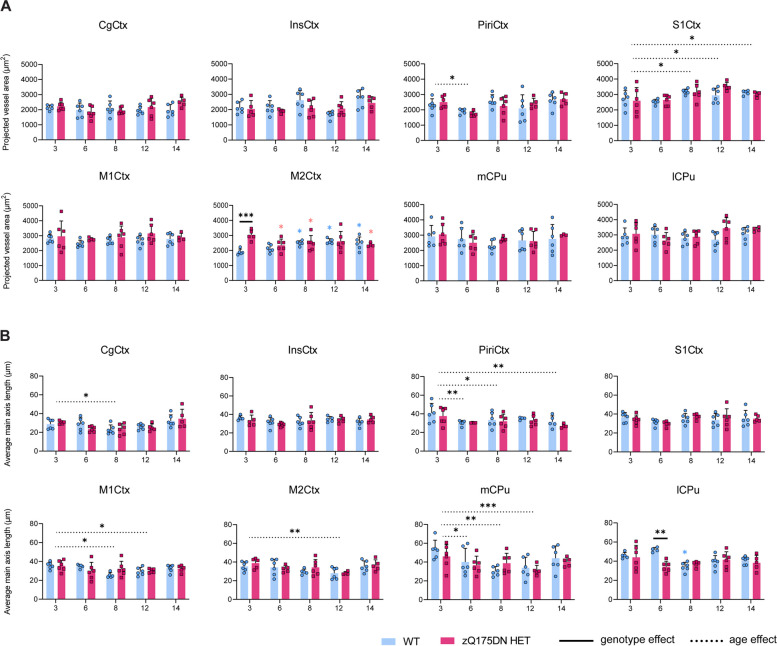
Blood vessel area and vessel length in the zQ175DN HET mice. (**A**) Projected vessel area (μm^2^) relative to FOV and (**B**) average main axis length (μm) in WT and zQ175DN HET mice at 3, 6, 8, 12 and 14 months of age for 8 brain regions, represented with subject values and a group mean ± SD (two-way ANOVA, *p* < 0.05); Colored asterisks represent the overall age effect within WT (blue) and zQ175DN HET (red), compared to 3 months. Significant difference after FDR correction * *p* ≤ 0.05, ** *p* ≤ 0.01, *** *p* ≤ 0.001, **** *p* ≤ 0.0001; CgCtx—Cingulate Cortex, InsCtx – Insular Cortex, Piri – Piriform Cortex, M1/2Ctx – Motor Cortex 1/2, S1Ctx – Somatosensory Cortex 1, CPu – Caudate Putamen, medial (mCPu) and lateral portion (lCPu)

Since perfusion and CVR changes have been shown to reflect changes linked to abnormal vascular structure and function in HD [42], we investigated vessel density and length in 8 brain regions in this model (Fig. 8). The projected vessel area showed only a significant genotype and age interaction in the M2Ctx (*p* = 0.0045), where post-hoc comparisons revealed a significant increase at 3 months of age in the zQ175DN HET group relative to WT (Fig. 8A). Moreover, compared to 3 months, within the zQ175DN HET group there was a significant decrease in vessel area for every age while in the WT, a significant increase in vessel area was present. A main effect of age was present in the S1Ctx, with an increase in vessel area in later ages, as compared to 3 months, while a significant decrease at 6 months is observed in the PiriCtx. When we assessed potential differences in the average length of the main vessel axis, we found a significant interaction only in the lCPu (*p* = 0.0094), where posthoc comparisons revealed a significant reduction in length at 6 months of age (Fig. 8B). A main effect of age was observed in the mCPu, M1Ctx, M2Ctx, PiriCtx and the CgCtx, where an overall decrease in main axis length, as compared to 3 months, was present.

## Discussion

Ours is the first study to characterize whole-brain, longitudinal changes in both brain perfusion at rest and vascular reactivity across disease progression in an HD animal model. At 3 months of age, a transient increase in resting-state CBF was present in the motor, somatosensory and retrosplenial cortices, followed by hypoperfusion in the piriform at 6 months and the somatosensory and retrosplenial cortex at 9 months of age in the zQ175DN HET mice. Under hypercapnia, multiple cortical regions showed insufficient increase of CBF at 6 months, whereas at 9 months, only the somatosensory and retrosplenial cortices displayed smaller increases in CBF, compared to controls. By 15 months of age, we observed a brain wide reduced CBF under hypercapnia. Vascular reactivity impairments were found at 9 months in several cortical areas as well as in the thalamus and globus pallidus, and at 15 months, where an overall diminished reactivity was present in the zQ175DN HET mice. Blood vessel density measurements demonstrated increased density in the motor cortex 2 at 3 months, while the average main vessel axis length revealed decreased length in the lateral caudate putamen at 6 months of age.

**Fig. 9:**
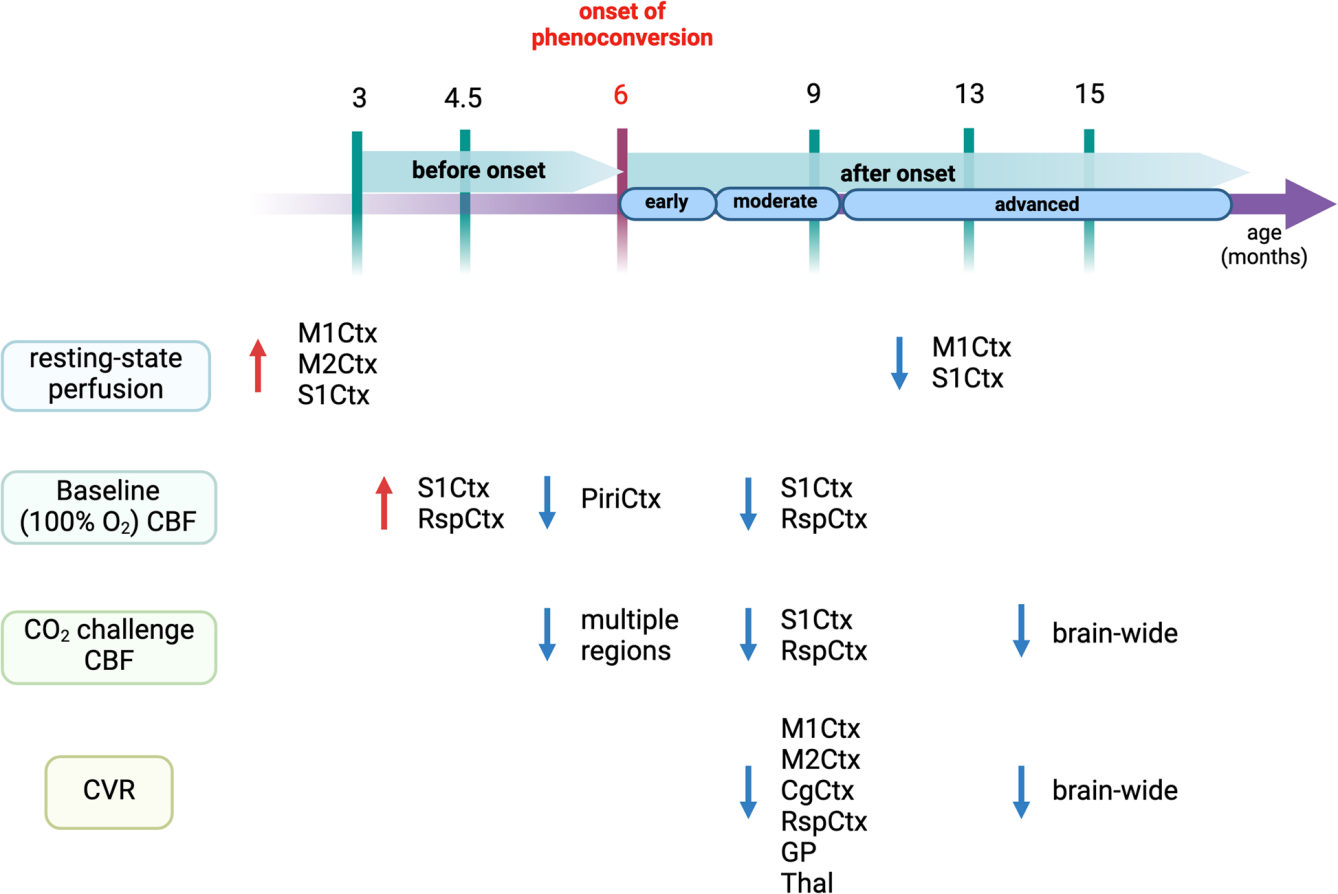
Summary of perfusion and vascular reactivity alterations along HD-like phenotypic progression in the zQ175DN HET mice. Created with Biorender.com

Increases in neuronal activation are tightly coupled to changes in CBF, cerebral blood volume (CBV) and blood oxygenation [43–45]. Our findings of increased cortical resting-state perfusion at 3 and 4.5 months of age could potentially be explained by an early hypermetabolic state of cortical neurons. Cortical hyperexcitability has been reported as an early event in several HD models [46–48]. Increased frequency of Ca^2+^ transients was observed in cortical pyramidal neurons of the M1Ctx at 2–3 months in the zQ175 model^47^, coinciding with our results where, at the same age, the M1Ctx showed hyperperfusion. When age- and region-specific cortical excitability were tested with ex-vivo electrophysiology in the zQ175 model, cortical pyramidal neurons in the somatosensory cortex showed biphasic changes with hyperexcitability states present at 3 – 4 months of age followed by hypo-excitability at 8 – 9 months of age [49]. Similarly, in our study, these biphasic changes were observed in the S1Ctx as well as the M1Ctx in resting-state and baseline perfusion at the same ages (Fig. 9). As functional hyperaemia underlies the well known concept of neurovascular coupling, a possible explanation would be that the cortical hyperaemia at the earliest ages is a consequence to increased excitability in these regions, as well as the hypo-excitability related reduced perfusion observed in later ages. Regional CBV, an important measure of brain metabolism, has been consistently shown to be altered in both HD models and PwHD before motor deficits occur [50, 51]. A longitudinal study of arteriolar CBV (aCBV) changes in the zQ175 mice has reported an increase in CBV in the motor cortex and striatum at 3 months of age before brain atrophy and behavioural changes were observed [51].

At 9 months of age, aCBV in the zQ175 HET mice was shown to be decreased in the motor cortex and striatum [51]. Here, we observed decreased resting CBF in cortical regions at 6 and 9 months, when both brain atrophy and motor impairments are detected [22, 51]. In PwHD, studies have reported cortical hypoperfusion without correlations to cortical atrophy post-clinical motor diagnosis [9, 18]. We did not observe changes in resting-state perfusion in the caudate putamen at any age. However, conflicting findings are reported in terms of perfusion-related alterations in the striatum in both clinical [9, 15, 19, 52] and preclinical studies [42, 51].

Evoked hemodynamic responses to specific external stimuli to test vascular delivery of nutrients under physiologically modified conditions have been seldom investigated in HD [10, 20]. Using a 10% CO_2_ challenge, we observed at 6 and at 15 months (the latter being an advanced age) a marked overall decrease in CBF in multiple cortical regions, the caudate putamen and globus pallidus in the zQ175DN HET mice. Reduced perfusion under hypercapnic conditions has also been reported in the R6/2 mice at 7 weeks of age, using a different MRI technique [42]. Evoked cortical dynamics responses, measured with local field potentials, after forelimb, auditory and visual stimulation have shown a broad increase in cortical sensory spread, in S1Ctx and MCtx, in the zQ175 mouse model at 6 months of age, indicative of aberrant cortical processing being present at the onset of motor anomalies [53]. As we observe significantly reduced perfusion under hypercapnia at 6 months in these regions as well, a plausible interpretation would be that neurovascular uncoupling or metabolic demand mismatch could occur due to altered vascular responses. Several studies have reported initial behavioural deficits and brain atrophy in the zQ175 model at 6 months of age [22, 23, 51], indicating that cognitive and motor abnormalities may start to occur as a result of an altered neurovascular network at this age that serves as a turnover point where compensatory mechanisms start to fail. The insular and piriform cortex, regions responsible for sensory integration and olfactory processing, show a significantly reduced CBF under hypercapnic conditions at this age (Fig. 6B). Interestingly, olfactory dysfunction has been implicated as an early event in PwHD [54, 55], and also in HD mouse models [56, 57], where especially in the zQ175 mice deficits in olfaction have been reported at an advanced phenotypic age [56]. At the age of 9 months, we found a significant decrease of both baseline and hypercapnic CBF in the RspCtx, region that plays a major role in multimodal sensory information processing and spatial navigation [58]. Even though the initial motor phenotype appears at 6 months, the most robust behavioural deficits are developed by 9 months in the zQ175 mice, implying that perfusion alterations could be causative of this robust phenotype [22, 59].

CVR impairments in PwHD have only been investigated in the past five years, where altered vascular responses have been reported in cortical and subcortical white matter regions at early stages post clinical motor diagnosis [10, 20]. The first and only preclinical study to assess vascular reactivity was in the R6/2 mouse model, where CVR impairments were observed in both the motor cortex and striatum at ages where motor deficits are present [42]. Here, we found altered CVR at 9 months, with decreased CVR in several cortical regions, the thalamus and the globus pallidus. At an advanced age of 15 months, we observed a globally altered CVR in the zQ175DN HET mice. Changes in the external globus pallidus neurons were already observed in this same model at 6–7 months of age, where hyperactivity was present in parvalbumin (PV)^+^ and hypoactivity in PV^−^ neurons [60]. One explanation for this could be a cell-specific dysfunction due to altered energy demands and metabolism in the globus pallidus in the zQ175 mouse model. Moreover, the overall pathology in this model is fully developed by 18 months [61], supporting the whole-brain impaired CVR near that age, as observed in our findings.

Multiple studies have reported that increased vascular density is present in PwHD after motor clinical diagnosis [17, 52] and in HD models at ages by which behavioural deficits are already present [42, 51, 52]. We assessed the blood vessel density in 8 brain regions across 5 ages in zQ175DN HET mice and found an increase in vessel density at 3 months of age in the M2Ctx, but not at later ages. Interestingly, the resting-state CBF was also increased in this region at the same age. The relevance of the role of M2Ctx in HD as a potential therapeutic target was reported in a study in R6/1 mice, where stimulation of projections from M2Ctx to the lateral caudate putamen rescued behavioral alterations and synaptic plasticity [62]. It could be that the augmented vessel density at this age could constitute a compensatory response to match the energy demands due to cortical hyperactivity that further results in hyperperfusion.

Several limitations should be considered in this study. First, we used only zQ175 HET male mice since they exhibit brain volume changes, resembling what is observed in PwHD [63]. Another consideration is the difference in baseline gas composition with respect to oxygen enrichment. In the resting-state CBF and baseline CBF in the CVR study, we used 33% and 100% O_2_ gas composition, respectively. Conversely, hyperoxia per se has been shown to not affect CBF or CBV measures, especially in mice where mean arterial pressure, respiratory rate and partial CO_2_ pressure are unchanged [64–66]. The overall use of ASL in rodents should be interpreted with caution since small brain size can lead to challenges in achieving comparable spatial resolution as well as signal-to-noise ratio. Moreover, these experiments were performed under anaesthesia and with different gas compositions, conditions that limit to an extend a direct translation to humans. Limitations concerning the IF study is that vessels are detected in 2D after maximum projection. A 3D analysis in a larger volume could give more detailed information. However, the projected area and vessel length are good initial indicators. The blood vessel IF study was performed using lectin as a marker, which binds to glycoproteins located in the basal membrane of endothelial cells, but it is nonspecific for vessel types. Markers for arterioles and capillaries should be employed in future studies to disentangle the specific vessel type that could contribute the most to the observed phenotype.

## Conclusion

Taken together, these findings reveal that cortical alterations in resting-state CBF occur as early events in the zQ175DN HET mice. Neurovascular network deficiencies are reflected under conditions of the evoked hemodynamic response, such as hypercapnia, where vascular reactivity impairments occur and progress in HD-like phenotypic states. This study puts forth brain perfusion and cerebrovascular dysregulation as important mechanisms that can contribute to the development of HD-like pathology.

## Data Availability

The datasets used and/or analysed during the current study are available from the corresponding author on reasonable request.

## Abbreviations

aCBV: Arteriolar cerebral blood volume
ANTs: Advanced normalization tools
ASL: Arterial spin labelling
CBF: Cerebral blood flow
CBV: Cerebral blood volume
CVR: Cerebrovascular reactivity
HD: Huntington’s disease
HET: Heterozygous
mHTT: Mutated huntingtin protein
pCASL: Pseudo-continuous arterial spin labelling
PwHD: People with Huntington’s disease
WT: Wild type
RARE: Rapid acquisition with refocused echoes
MD: Matrix dimensions
FOV: Field of view
PLD: Post-labelling delay
SE-EPI: Spin echo planar imaging
IR: Inversion recovery
TI: Inversion times
SPM: Statistical parametric mapping
RBA: Region-based analysis
VBA: Voxel-based analysis
ROI: Region of interest
PoC: Proof-of-concept
mCPu: Caudate putamen, medial portion
lCPu: Caudate putamen, lateral portion
CgCtx: Cingulate cortex
InsCtx: Insular cortex
PiriCtx: Piriform cortex
S1Ctx: Somatosensory cortex 1
M1Ctx: Motor cortex 1
M2Ctx: Motor cortex 2
RspCtx: Retrosplenial cortex
Thal: Thalamus
GP: Globus pallidus
FDR: False discovery rate
